# Insideout project: Using big data and machine learning for prevention in psychiatry

**DOI:** 10.1192/j.eurpsy.2021.919

**Published:** 2021-08-13

**Authors:** F. Fiori Nastro, D. Croce, S. Schmidt, R. Basili, F. Schultze-Lutter

**Affiliations:** 1 Department Of Systems Medicine, University of Rome “Tor Vergata”, Roma, Italy; 2 Department Of Enterprise Engineering, University of Rome “Tor Vergata”, Roma, Italy; 3 Clinical Psychology And Psychotherapy, University of Bern, Bern, Switzerland; 4 Department Of Enterprise Engineering, University of Rome “Tor Vergata”, Rome, Italy; 5 Department Of Psychiatry And Psychotherapy, Heinrich-Heine-University, Düsseldorf, Germany

**Keywords:** distress, prevention, machine learning, e-mental health

## Abstract

**Introduction:**

Social Media might represent an amazing and valuable source of information on mental health and well-being. Several researches revealed that adolescents aged 13 to 17 years old go “online” daily or stay online “almost constantly”.

**Objectives:**

The aim of this project is to identify distress in pre-clinical stages using Social media screening methods. The system can be modelled to centre on different several health-related topics.

**Methods:**

We created a digital system able to analyse scripts written by adolescents on Twitter. InsideOut works using machine learning techniques and computational linguistic items to catch significant and sense of written messages and it improves its performances with iterations. The system is able to automatically identify semantic information relevant to different topics: in this case “distress in teenagers”.

**Results:**

The task of our system is considered correct when it is able to identify triples of Life Event, Sentiment and Experience of a tweet in agreement with the Gold Standard established among the annotators. The system has around 70% of accuracy in identifying triples. The analysis has been carried out both in Italian and English collecting over 4 million Italian tweets and 30 million English tweets. Comparative analysis with self-report questionnaires show that tweet analysis is able to suggest similar statistics.

**Conclusions:**

This study analyzed contents of messages posted on Social Media Twitter meta-dating them with psychological and health-related information. Using InsideOut, we can plan clinical intervention in district and regions where high levels of uneasiness are revealed.

